# Investigation of the Genetic Association between Quantitative Measures of Psychosis and Schizophrenia: A Polygenic Risk Score Analysis

**DOI:** 10.1371/journal.pone.0037852

**Published:** 2012-06-22

**Authors:** Eske M. Derks, Jacob A. S. Vorstman, Stephan Ripke, Rene S. Kahn, The Schizophrenia Psychiatric Genomic Consortium, Roel A. Ophoff

**Affiliations:** 1 Rudolf Magnus Institute of Neuroscience, Department of Psychiatry, University Medical Center Utrecht, The Netherlands; 2 Department of Psychiatry, Academic Medical Center, University of Amsterdam, Amsterdam, The Netherlands; 3 Center for Human Genetic Research, Massachusetts General Hospital, Boston, Massachusetts, United States of America; 4 UCLA Center for Neurobehavioral Genetics, Semel Institute for Neuroscience and Human Behavior, Los Angeles, California, United States of America; 5 Department of Medical Genetics, University Medical Center Utrecht, Utrecht, The Netherlands; University of Iowa Hospitals & Clinics, United States of America

## Abstract

The presence of subclinical levels of psychosis in the general population may imply that schizophrenia is the extreme expression of more or less continuously distributed traits in the population. In a previous study, we identified five quantitative measures of schizophrenia (positive, negative, disorganisation, mania, and depression scores). The aim of this study is to examine the association between a direct measure of genetic risk of schizophrenia and the five quantitative measures of psychosis. Estimates of the log of the odds ratios of case/control allelic association tests were obtained from the Psychiatric GWAS Consortium (PGC) (minus our sample) which included genome-wide genotype data of 8,690 schizophrenia cases and 11,831 controls. These data were used to calculate genetic risk scores in 314 schizophrenia cases and 148 controls from the Netherlands for whom genotype data and quantitative symptom scores were available. The genetic risk score of schizophrenia was significantly associated with case-control status (p<0.0001). In the case-control sample, the five psychosis dimensions were found to be significantly associated with genetic risk scores; the correlations ranged between.15 and.27 (all p<.001). However, these correlations were not significant in schizophrenia cases or controls separately. While this study confirms the presence of a genetic risk for schizophrenia as categorical diagnostic trait, we did not find evidence for the genetic risk underlying quantitative schizophrenia symptom dimensions. This does not necessarily imply that a genetic basis is nonexistent, but does suggest that it is distinct from the polygenic risk score for schizophrenia.

## Introduction

In clinical practice as well as in most research schizophrenia is conceptualized as a categorical entity, allowing for a distinction in the population between affected and unaffected. Yet, it has been proposed that psychotic symptoms, in essence the same as those observed in individuals with schizophrenia, can also be measured at subclinical levels in individuals without schizophrenia in the general population [1]. The concept of a psychosis continuum implies that schizophrenia is not a categorical disorder, but rather the extreme expression of otherwise more or less continuously distributed traits in the population [2]. This view has gained momentum in recent years. For instance the results of a meta-analysis showed prevalence rates of psychotic experiences and symptoms of approximately 4–8% in the general population. In addition, some of the previously identified risk factors for schizophrenia, including cannabis, traumatic experiences and urbanicity, also increase the risk of psychotic experiences in the non-clinical population [3]. A recent study demonstrated that, using affectedness of relatives as a proxy, a higher genetic loading increased the risk of psychotic symptoms in subjects without a clinical psychotic disorder [4]. To date, no study has explored a possible correlation between a direct continuous measure of genetic risk and the continuous psychosis phenotype. The question is not trivial; finding such correlation would provide strong genetic evidence for the concept of the psychosis continuum. Inversely, the demonstration of such correlation would provide a strong argument for investigating the dimensional scale of psychotic experiences or symptoms in genetic studies [5].

Reporting on data from the International Schizophrenia Consortium, Purcell and colleagues presented evidence in support of a polygenic contribution to schizophrenia [6]. They demonstrated that the available Genome Wide Association (GWA) findings are compatible with a large number of shared loci each with very small odds ratios contributing to disease susceptibility. Based on the nominally associated alleles in a discovery sample, a quantitative polygenic risk score was calculated. Subsequently, when comparing this polygenic risk score between cases and controls in two independent schizophrenia samples a significantly higher signal was detected in cases. As was proposed recently by Plomin and colleagues, the involvement of multiple genes indicated by the GWAS results for a disorder suggests that the genetic liability may be distributed quantitatively rather than qualitatively. This in turn raised the question to which extent this distribution of polygenic liability is mirrored in a similar distribution of quantitative traits which compose the disorder [7].

Interestingly, the schizophrenia-derived polygenic risk score was also shown to be significantly increased in bipolar disorder [6]. This could be interpreted as an indication that genetic liability can be present with regard to certain symptom domains, rather than for a specific (categorically defined) syndrome of symptoms. In other words, it can be argued that the liability for schizophrenia is composed of co-occurring different genetic liabilities for different symptom domains. This was supported by recent findings suggesting the presence of two distinct polygenic risk scores: one that relates to expression of a 'bipolar disorder-like' phenotype and one that is associated with expression of 'schizophrenia-like' psychotic symptoms [8].

Several studies have investigated whether genetic factors contribute to symptom dimensions of schizophrenia. Results of twin studies suggest heritability of symptoms of disorganization [9] and reality distortion, i.e. hallucinations and/or delusions [10]. A meta-analysis of the results of studies on symptom concordance in schizophrenia affected sibling pairs demonstrated significant correlations within siblings for the dimensions psychomotor poverty, reality distortion and disorganization, with the latter showing the highest correlation coefficient [11]. Individual genes have also been suggested to differentially impact the different quantitative symptom dimensions (reviewed in [12], [13]).

We recently performed factor analyses on 79 symptoms related to schizophrenia in a large sample of over four thousand subjects, approximately half of which were healthy controls, while the other half of the subjects were diagnosed with affective or non affective psychotic disorders or non psychotic mood disorders [14]. This was a first attempt to detect latent dimensions for schizophrenia in a sample that included both psychotic and non-psychotic patients as well as healthy controls. Our analyses indicated five continuous dimensions of schizophrenia: positive, negative, disorganisation, mania, and depression. Importantly, these results have provided us with measures that express five phenotypic components of the schizophrenia phenotype as continuous, quantitative traits.

The current study was set up to examine the correlation between a direct measure of genetic risk of schizophrenia, using the polygenic risk score, and quantitative measures of schizophrenia symptoms, using the five continuous symptom dimensions of schizophrenia derived from our previous study. We hypothesize a positive correlation between the polygenic risk score and one or more of the symptom dimension scales in individuals with and without a diagnosis of schizophrenia.

We propose that if a significant correlation can be confirmed, this finding would provide genetic evidence for the psychosis continuum concept by substantiating the notion proposed by Plomin et al., namely that the polygenic liability is mirrored in a similar distribution of quantitative traits which compose the disorder [7].

## Methods

### Subjects

From a sample of 715 schizophrenia cases and 643 controls from The Netherlands, genotypic data of 704 cases and 631 controls passed Quality Control (QC) criteria. We additionally removed 10 subjects (5 cases and 5 controls) who were indicated as outliers according to a principal component analysis performed in EIGENSOFT [15]. This resulted in the final case-control sample including data of 699 cases and 626 controls. Detailed phenotypic assessments (i.e., the Comprehensive Assessment of Symptoms and History (CASH) [16]) was collected in a smaller subsample including 314 schizophrenia patients and 148 controls. This is the sample that was used for the analyses of the symptom dimension scores. The controls had no history of psychiatric disorder. All patients and controls had at least three grandparents of Dutch ancestry. The study was approved by the institutional ethical committee and informed consent was obtained from all participants. A more detailed description of the inclusion protocol has been described elsewhere [17].

### Genotyping and Quality Control Procedure

All genome-wide genotyping for the GWAS was performed on Human- Hap550v3 BeadArrays using the Infinium II assay (Illumina) at the Southern California Genotyping Consortium (SCGC) at UCLA, Los Angeles, USA.

An extensive quality control (QC) protocol was carried out, the procedure has been described in full detail [18]. Briefly, SNPs were included if the missing rate was <.02, the SNP frequency difference to HapMap was <.15, the difference missing rate per SNP between cases and controls <.02), and Hardy-Weinberg Equilibrium was not violated in controls (p<10^−6^). Individuals were included if the missing rate was <.02. We removed one member of a pair of observations in case of duplication or cryptic relatedness. Finally, ten subjects were indicated as outliers according to the principal component analysis and were removed from subsequent analyses. The QC protocol resulted in a sample of 699 cases and 626 controls. The genomic inflation factor of this sample was 1.02; the QQ plot is shown in Figure S1.

### Statistical Analysis

We have previously calculated liability scores on the five psychosis dimensions (i.e., disorganization, positive, negative, mania, and depression) in a confirmatory factor analysis including CASH lifetime rated symptoms from 4,286 subjects. Of these individuals, N = 1,965 were healthy controls while the remaining individuals were diagnosed with a psychiatric disorder (N = 1,085 schizophrenia or schizophreniform disorder; N = 160 schizoaffective disorder; N = 202 bipolar disorder; N = 480 major depression; N = 388 other psychiatric diagnoses) [14]. The subjects included in the present study are a subset of this larger sample.

Estimates of the log of the odds ratios of case/control allelic association tests were obtained from the Psychiatric GWAS Consortium (PGC) sample (but excluding the Utrecht/UCLA sample) which included Single Nucleotide Polymorphism (SNP) data (N = 1,241,601) from 8,690 schizophrenia cases and 11,831 controls. SNPs were imputed with HapMap-3 [19] as the reference panel; confidence metrics in the single datasets were set at 0.1.

We selected SNPs which were associated with case-control status below a fixed p-value. Three selection thresholds were applied; all SNPs associated at p<.5 (selection 1); all SNPs associated at p<.1 (selection 2); and all SNPs associated at p<.01 (selection 3). LD pruning was applied to select SNPs which are in approximate linkage equilibrium with each other. We used the –indep option in PLINK with the default values for the parameters (i.e., window size of 50 kb, the number of SNPs to shift the window at each step = 5, and a Variance Inflation Factor (VIF) threshold of 2 [20]. The total number of SNPs in the three analyses was 63,935 (selection 1; p<.50); 14,654 (selection 2; p<.10); 1,954 (selection 3; p<.01).

Genetic risk scores were calculated in PLINK [20] using the method described by Purcell and colleagues [6].

**Table 1:** Association of genetic risk scores with symptom dimensions and case-control status across thresholds.

	Pearson correlations between genetic risk scores and symptom dimensionsin the total sample and by status					Case-controlstatus
	Positive Total(case/control)	Negative Total(case/control)	Disorganisation Total(case/control)	Mania Total(case/control)	Depression Total(case/control)	Mean cases/mean controls
Genetic risk score: p<.5	.15*(−.01/.06)	.19**(.03/.07)	.09 (−.09/.05)	.12* (−.05/.02)	.16** (−.06/.06)	.13/−.18**
Genetic risk score: p<.1	.17**(.03/.05)	.17**(.03/.08)	.08(−.10/.08)	.12(−.07/.08)	.15*(−.06/.08)	.14/−.17**
Genetic risk score: p<.01	.13*(.06/.03)	.13*(.04/.02)	.03(−.08/−.09)	.07(.01/−.06)	.11(.03/−.08)	.09/−.12**

** p<.001.

* p<.01.

Briefly, risk scores were calculated based on an individual's genotype. For each SNP, the log of the odds ratio of an allele was multiplied by (0, 1, or 2) depending on the number of risk alleles that an individual carries. The total polygenic risk score is simply a sum across SNPs.

In the subsequent analyses, we aimed to correct for the possible presence of population stratification, by adjusting for the first 10 principal components which were calculated with EIGENSOFT [15]. A logistic regression analysis was used to investigate whether the genetic risk score is indeed significantly associated with case-control status in our independent sample to replicate the results of Purcell et al. [6]. Nagelkerke R squared was used to compare the percentage of variance in case-control status explained by the first 10 principal components with the percentage of variance explained by the first 10 principal components and the genetic risk score.

Next, we calculated partial correlations between genetic risk scores (including all SNPs associated at p<.5) and dimension scores, adjusting for the first 10 principal components in a sample of 314 schizophrenia cases and 148 controls. This analysis was performed in the total sample and in cases and controls separately. Power analyses performed in statistical package R [21] demonstrated that within cases and controls this study had 80% power to detect correlations of.22 and.16 respectively, using a type-I error rate of 5%. We also repeated the case-control analysis in this smaller subsample to facilitate comparison of the results of the case-control analysis and the dimension score analyses.

**Figure 1a–e:**
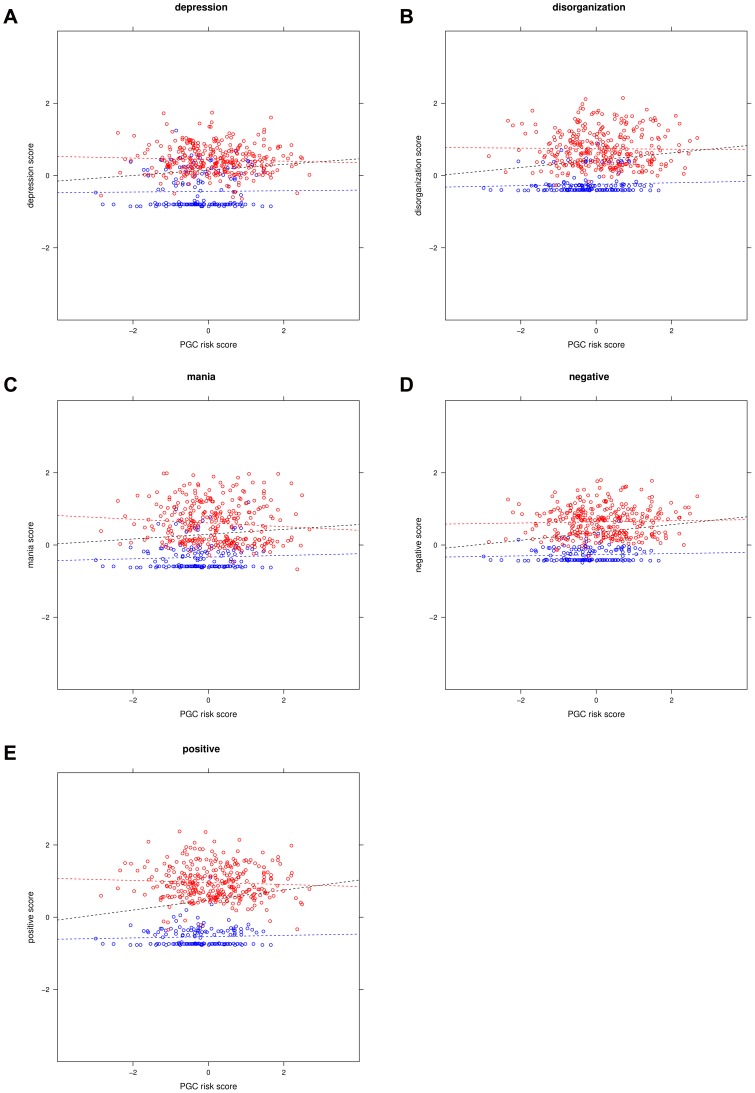
e. Associations between symptom dimension scores and genetic risk scores. Legend “The red dots represent cases and the blue dots represent controls. The regression lines represent the association between genetic risk score and dimension scores in the total sample (black) in cases (red) and in controls (blue).

## Results

A graphical representation of the distribution of the genetic risk scores is provided in Figure S2 for cases and controls respectively.

The polygenic risk scores were standardized (i.e., mean = 0 and SD = 1) in the total sample. A summary of mean genetic risk scores by case/control status is provided in Table 1 for each of the three selections. The logistic regression analysis indicated that the 10 principal components explained 1.5% of the variance in case/control status (X^2^(10) = 15.45, p = .12). The prediction of case-control status significantly improved by including the genetic risk factor as a predictor with the proportion of variance increasing to 2.9%, 4.7%, and 5.1% based on the inclusion of SNPs associated at p<.01, 0.10, and 0.50, respectively. Substracting the variance explained by the principal components, this implies that ∼1.4 to 3.6% of the variance in case-control status is explained by the genetic risk score. The association between genetic risk score and case-control status was highly significant. For example, using a threshold of p<0.50, the regression coefficient of the genetic risk score was 0.36 (Wald = 5.89, p<0.0001). Including all SNPs associated at p<0.50, the mean genetic risk scores were.13 (SD = .98) and -.18 (SD = .94) in cases and controls, respectively (see Figure S2). Exclusion of the SNPs in the extended MHC region (6p21.31–6p22.1) did not affect the results.

Next, we studied the association between psychosis dimensions and genetic risk scores (see Table 1). In the total sample of cases and controls, the correlations between psychosis dimensions and genetic risk scores range between.09 and.19. These correlations were statistically significant (p<.01) for the dimensions positive (r = .15), negative (r = .19), mania (r = .12), and depression (r = .16) while the correlation of the dimensions disorganization (r = .09) was not statistically significant (p = .04). The positive correlations are not unexpected since case-control status is associated both with dimension scores and genetic risk scores. Therefore, we continued our analyses by testing whether genetic risk scores are significantly associated with psychosis dimension scores in the cases and controls separately. In schizophrenia cases, the correlations ranged between −.06 and.04 and were not significantly different from zero (all p>.10). Similarly, the correlations in controls were not significant; the range was between.02 and.07 (all p<.10).

Figure 1a–e show the associations between genetic risk scores and psychosis dimensions in the total sample with cases and controls plotted in different colors. As dimension scores were assessed in a relatively small subsample (24% of the controls and 45% of the cases), we repeated the case-control analysis in this subsample to facilitate comparison of the results. Including all SNPs associated at p<.50, 7% of the variance was explained by the 10 principal components (X^2^(10) = 22.66, p = .01) which increased to 13% after inclusion of the genetic risk score (X^2^(11) = 43.23, p<.0001).

## Discussion

The aim of this study was to investigate the correlation between the polygenic liability for schizophrenia and quantitative domains of schizophrenia symptoms in schizophrenia cases and healthy controls. In the current sample, we replicate the findings by Purcell et al. [6] and show that the polygenic risk score effectively predicts schizophrenia status in our sample. Given that both the dimension scores and the genetic scores are highly associated with case-control status it is not surprising that the polygenic score was also significantly correlated with each of the five schizophrenia dimensions when analyzing the entire sample. The polygenic risk score did not have a significant correlation with any of the five symptoms dimensions when cases and controls were analyzed separately. Therefore, we conclude that the genetic basis of severity differences within diagnostic subgroups (i.e., cases vs. controls) is not shared with the genetic basis of case-control status.

There are several possible explanations for this finding. First, in reality such genetic basis exists but the score alleles (SNPs) used in the current study do not index severity of the schizophrenia dimensions selected in the current study. SNPs were selected based on their association with case-control status and possibly, other score alleles may be correlated with the schizophrenia dimensions used in the current study. It is also possible that the used score alleles may be correlated with schizophrenia dimensions other than those used in our study. Alternatively, the explained variance is very small with correlations <.2, which are potentially not detected due to a lack of statistical power given the sample size of this study. A whole different explanation may be that while there is a continuously distributed genetic liability correlated with schizophrenia dimensions, this genetic liability is not based on common allelic (SNP) variants. For instance, a continuously distributed measure based on rare genetic variants or on epigenetic variation is possible in theory. The most dramatic explanation for our observation that the schizophrenia polygenic risk score does not predict severity of symptom dimensions could be the absence of a continuously distributed genetic liability that explains the observed psychosis continuum. Please note that this is not inconsistent with the available evidence indicating a genetic contribution to schizophrenia dimensions; results so far [11] do not provide evidence that such genetic contribution is present when adjusting for case-control status.

It should also be noted that, based on the results of our previous study [14], we have chosen for the inclusion of five symptom dimensions. The results of factor analyses largely depend on the content of the items that are included. If we would have included items of additional instruments, the number and interpretation of the resulting factors could have been different. The results of factor analytical studies have been discussed by Peralta and Cuesta [22] who showed that the number of factors ranged from 4 to 11, depending on the content of the items included in the analyses. The inclusion of additional factors (e.g., psychomotor poverty) could result in different findings and we hope that other research groups, who have used different instruments for the assessment of psychosis will address this question. Future collaborative studies should aim to further elucidate the genetic basis of quantitative symptom dimensions by combining symptom ratings assessed in psychiatric cases (e.g., schizophrenia, bipolar disorder, depression), and healthy controls.

## Supporting Information

Figure S1 **QQ plot of the UCLA case-control sample.** This figure plots the expected –log_10_ (p) at the x-axis and the observed –log_10_ (p) at the y-axis.
(DOC)

Figure S2 **Distribution of genetic risk scores in schizophrenia cases and controls.** This figure shows the distribution of the genetic risk score in cases and controls.
(DOC)
