# Correction: Investigation of the Genetic Association between Quantitative Measures of Psychosis and Schizophrenia: A Polygenic Risk Score Analysis

**DOI:** 10.1371/annotation/6ff0353a-cc91-4d12-896a-d1de0dcb0fe0

**Published:** 2013-03-11

**Authors:** Eske M. Derks, Jacob A. S. Vorstman, Stephan Ripke, Rene S. Kahn, The Schizophrenia Psychiatric Genomic Consortium, Roel A. Ophoff

The Supporting Information files in the paper do not appear properly. The correct files can be found here:

Figure S1: 

Figure S2: 
