# Supplementary material for: Investigation of the Genetic Association between Quantitative Measures of Psychosis and Schizophrenia: A Polygenic Risk Score Analysis
Source: PLoS One. 2012 Jun 22;7(6):e37852. doi: 10.1371/journal.pone.0037852 (PMC3382234; doi:10.1371/journal.pone.0037852)
Supplement: Supplementary file 1 [file pone.0037852.s001.doc]

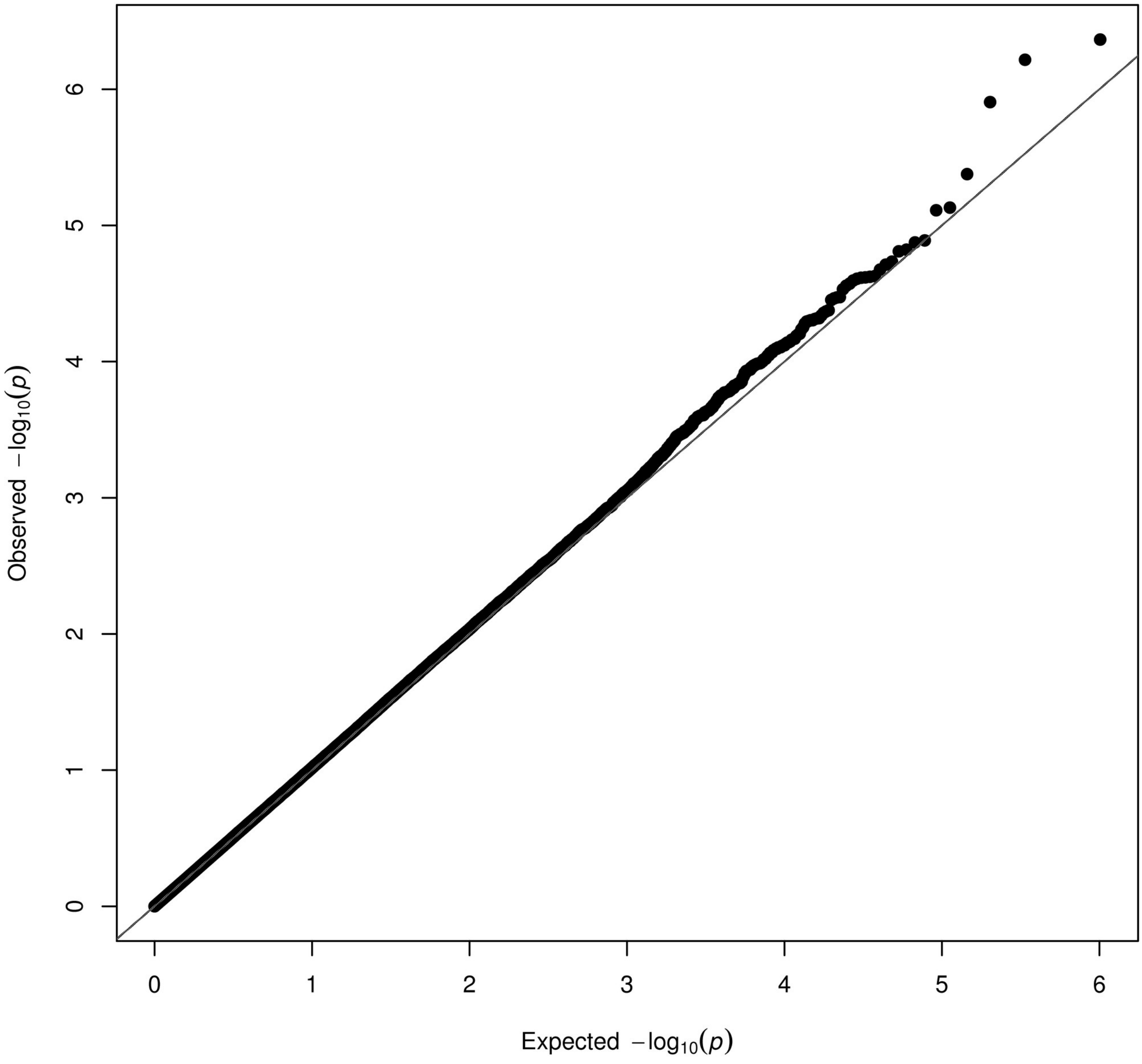

Observed $-\log_{10}(p)$
Expected $-\log_{10}(p)$
0
1
2
3
4
5
6
0
1
2
3
4
5
6
