# Supplementary material for: Investigation of the Genetic Association between Quantitative Measures of Psychosis and Schizophrenia: A Polygenic Risk Score Analysis
Source: PLoS One. 2012 Jun 22;7(6):e37852. doi: 10.1371/journal.pone.0037852 (PMC3382234; doi:10.1371/journal.pone.0037852)
Supplement: Supplementary file 2 [file pone.0037852.s002.doc]

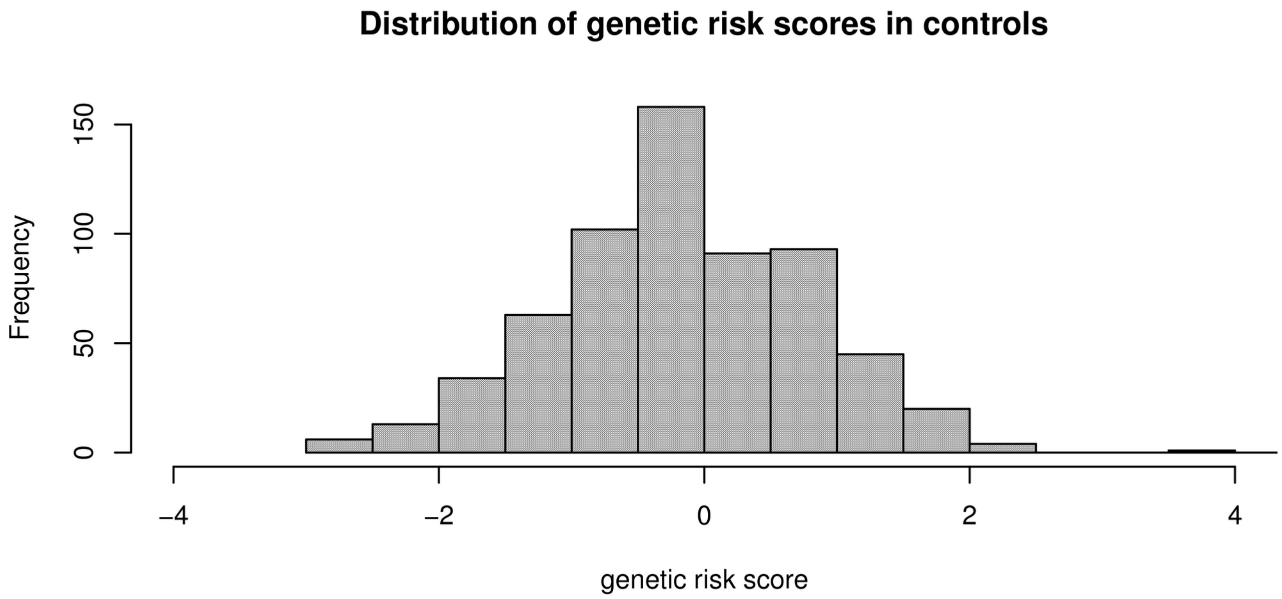

Distribution of genetic risk scores in controls
Frequency
0
50
100
150
–4
–2
0
2
4
genetic risk score

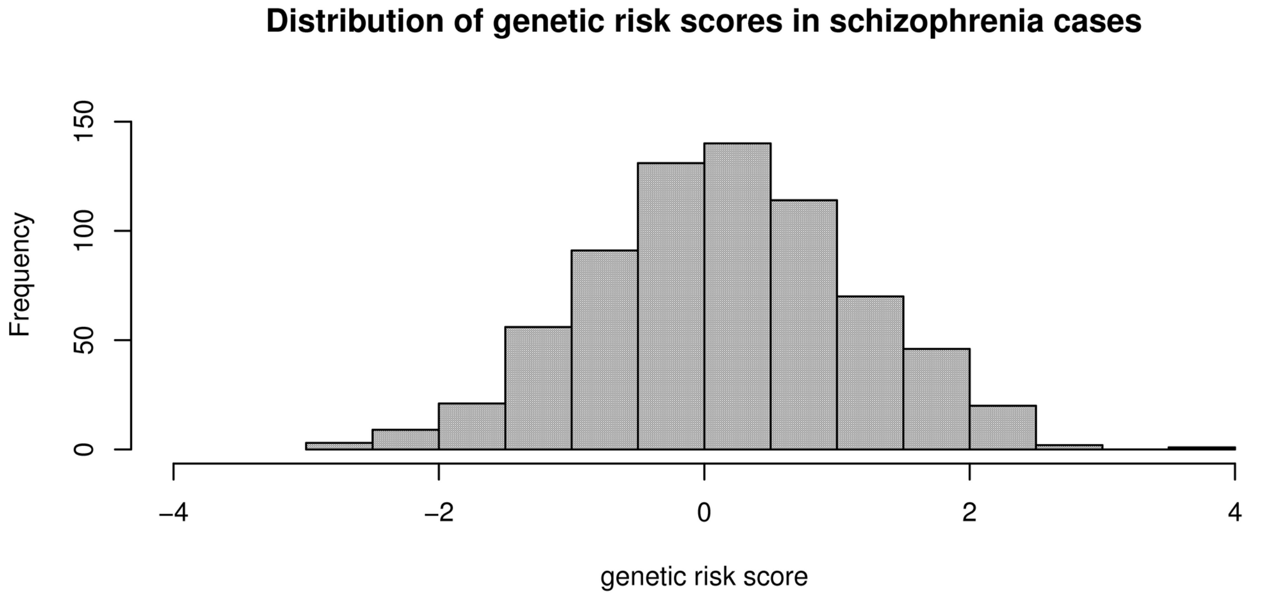

Distribution of genetic risk scores in schizophrenia cases
Frequency
0
50
100
150
–4
–2
0
2
4
genetic risk score
