# Supplementary figures and images for: Correction: Investigation of the Genetic Association between Quantitative Measures of Psychosis and Schizophrenia: A Polygenic Risk Score Analysis
Source: PLoS One. 2013 Mar 11;8(3):10.1371/annotation/6ff0353a-cc91-4d12-896a-d1de0dcb0fe0. doi: 10.1371/annotation/6ff0353a-cc91-4d12-896a-d1de0dcb0fe0 (PMC3600080; doi:10.1371/annotation/6ff0353a-cc91-4d12-896a-d1de0dcb0fe0)

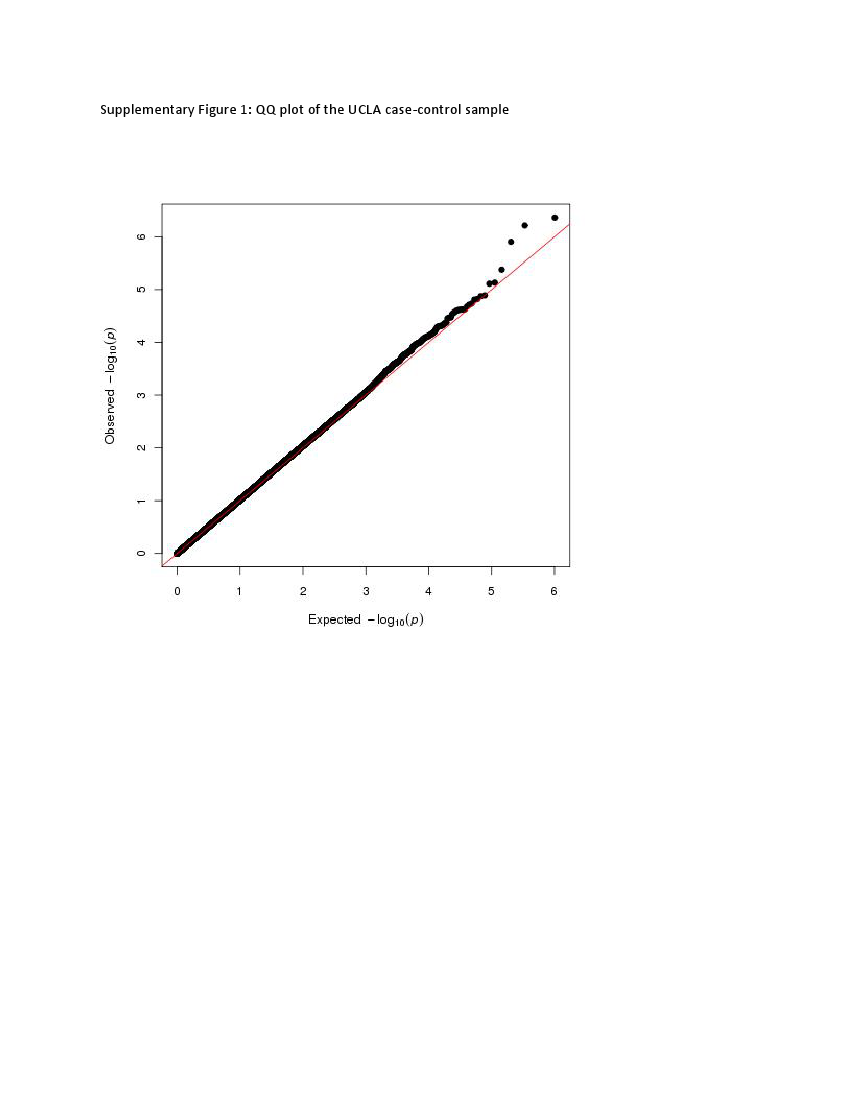

Supplement: Supplementary file 1 [file pone.6ff0353a-cc91-4d12-896a-d1de0dcb0fe0.s001.tif]

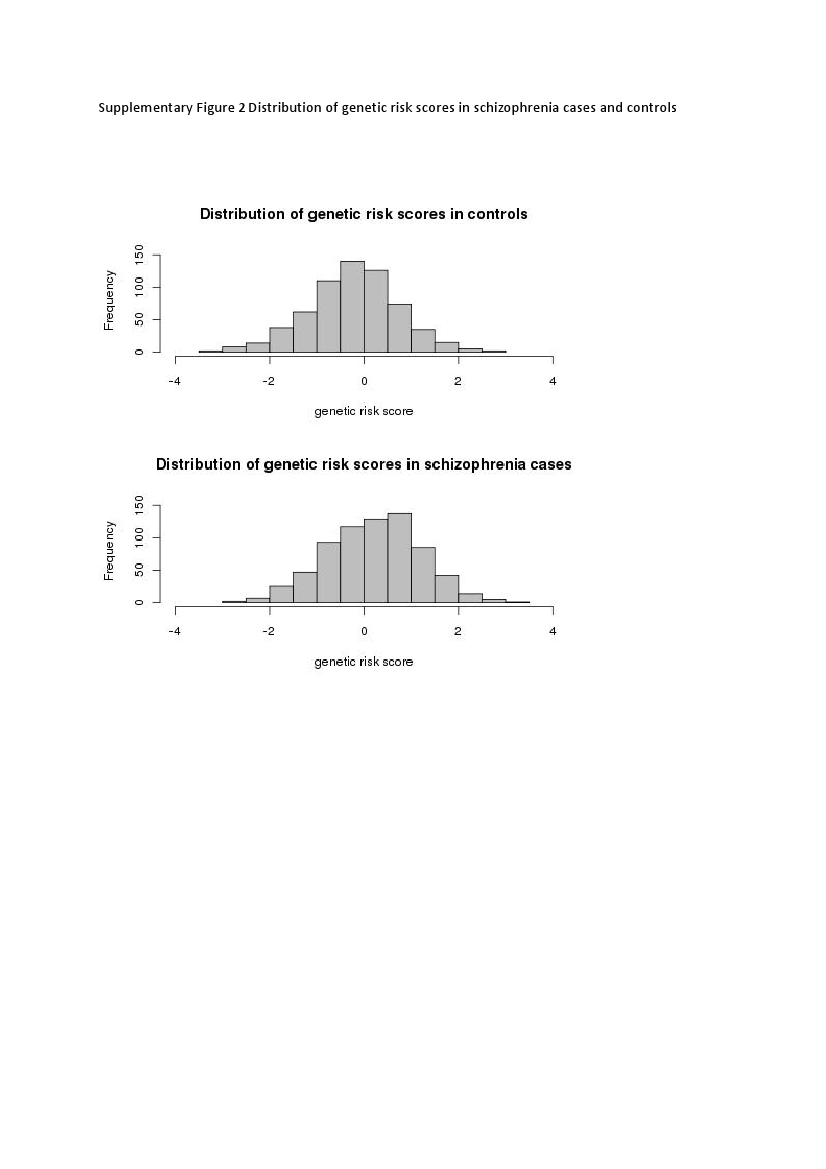

Supplement: Supplementary file 2 [file pone.6ff0353a-cc91-4d12-896a-d1de0dcb0fe0.s002.tif]
